# Monitoring and evaluation of lymphatic filariasis interventions: an improved PCR-based pool screening method for high throughput *Wuchereria bancrofti* detection using dried blood spots

**DOI:** 10.1186/1756-3305-6-110

**Published:** 2013-04-18

**Authors:** Catherine Plichart, Aurore Lemoine

**Affiliations:** 1 Institut Louis Malardé, Papeete, Tahiti, French Polynesia

**Keywords:** Lymphatic filariasis, Disease monitoring, Real-time PCR, Pool screen, Blood spots, French Polynesia

## Abstract

**Background:**

Effective diagnostic tools are necessary to monitor and evaluate interruption of Lymphatic Filariasis (LF) transmission. Accurate detection of *Wuchereria bancrofti* (*Wb)* microfilaria (mf) is essential to measure the impact of community treatment programmes. PCR-based assays are specific, highly sensitive tools allowing the detection of *Wuchereria bancrofti* DNA in human blood samples. However, current protocols describing the pool screening approach, use samples of less than 60 μl of blood, which limits the sensitivity of the pool-screen PCR assay. The purpose of this study was to improve the pool-screen PCR protocol to enhance its sensitivity and usefulness for population scale studies.

**Findings:**

DNA extractions were performed with the DNeasy kit, the PCR with the *Wb* LDR primers and the SYBR-Green dye. Improvements of our pool-screen real-time PCR (qPCR) assay allowed the detection of as little as one *Wb* microfilaria diluted in a pool of at least 12 blood samples of 60 μl each. Using this assay, mf burdens can be predicted using a standard curve derived from mf spiked dried blood samples. The sensitivity achieved is equivalent to the detection of a single LF positive individual carrying a mf burden as low as 18 mf/ml, in a pool of blood samples from at least 12 individuals.

**Conclusions:**

Due to its sensitivity, rapidity and cost-effectiveness, we suggest this qPCR pool-screening assay could be used as a diagnostic tool for population- scale filariasis elimination monitoring and evaluation.

## Findings

### Background

The main goal of the Global Program to Eliminate Lymphatic Filariasis (GPELF) is to interrupt disease transmission by reducing microfilaremia prevalence in blood through mass drug administration (MDA) [1]. Diagnostic tools are required to assess the status of LF in countries that are now in the post-MDA surveillance phase or are still implementing preventive chemotherapy. The rapid immunochromatographic test (ICT), detecting filarial antigen, is the selected tool for deciding when to stop MDA [2] in areas where levels of infection have been reduced to a point where transmission is no longer sustainable [3]. However, because antigenemia decreases more slowly than microfilaremia [4-6], the detection of mf in human blood populations of sentinel and spot-check sites remains an essential complementary test for assessing the impact of MDA [3,7-9]. Several methods are available for mf testing: the counting chamber method [10], the microscopic examination of capillary blood films (60-μl thick) [11-14] or membrane filtration from one ml of venous blood [15]. Because these techniques can only be realized on individual samples, they are labour intensive and not adapted for monitoring residual mf prevalence rates in the community. As mf prevalence decreases through MDA, the pool screening approach becomes necessary and more cost effective [8]. PCR assays have been developed [16,17] that are highly sensitive and specific for the detection of *Wb* DNA in individual human blood samples [18-20] as well as in mosquito vectors [21-25]. Conventional PCR and qPCR-based pool-screening methods, using pools of up to 10 blood samples of 10 μl to 30 μl each, have already been described [2,26-28]. However, they are less sensitive than the thick blood film method usually used [2], due to the low volume of blood analysed per patient. Thus, improving the pool-screening sensitivity of the qPCR assay by increasing the volume of blood tested would allow the broader implementation of this diagnostic tool.

The purpose of this study was to improve the DNA extraction protocol and qPCR assay to achieve the detection of a single mf in a pool of at least twelve 60-μl blood samples.

### Human blood samples

The assay was performed using capillary blood dried on filter paper as capillary blood is used to test microfilaremia in most countries endemic for LF. All procedures were carried out in the laboratory. All samples were acquired under protocols approved by the French Polynesia Ethics Committee with written informed consent obtained from all subjects. For each subject, 60 μl of blood was loaded on 6 spots of a filter paper disk (TropBio, Townsville, Australia), dried and stored at −20°C until use. Fifty-eight samples negative for filarial antigen (ICT and Og4C3) and for microfilariae (PCR and blood smear) were used for the assays. Samples containing one mf were obtained by very carefully spiking a single mf purified from the blood of a microfilaremic individual, onto the filter paper disk of a non-infected subject as identified above. Samples containing 3 mf and 10 mf were obtained similarly. Positive samples of approximately 100 mf were prepared with 60 μl of blood from an infected person whose microfilaremia was estimated at 1548 mf/ml by the filtration method. The six blood filter spots of each sample were processed.

### DNA purification

DNA was extracted from the blood filter spots using the QIAGEN DNeasy kit (Qiagen, Hilden, Germany; Cat N° 69504) following the manufacturer’s instructions and increasing the volumes of reagents [Laney SJ, unpublished data]. Briefly, blood spots of a subject were placed in a 2 ml microtube, covered with 270 μl of ATL buffer, incubated at 85°C for ten minutes, then at 56°C for one hour after addition of 30 μl of Proteinase K. Addition of 300 μl of Al buffer to the digested suspension brought up the lysate volume to a total of 600 μl. From this step, we used two different protocols. First, the lysate was either heat-treated (100°C) or not, prior to the DNA purification. The aim of the heat treatment was to denature the genomic DNA to make the DNA target sequence more accessible to the primers. Second, individual lysates (total or fraction) or pooled lysates were processed in the subsequent DNA purification step. A volume of ethanol equivalent to half the volume of lysate sample was added before loading the mix onto a DNeasy spin column. Depending on the volume loaded, additional centrifugations were performed to pass all the solution through the column. After washing steps (twice with AW1 buffer, once with AW2 buffer), purified DNA was eluted in 200 μl of AE buffer.

### Real-time PCR

qPCR assays were performed on the Bio-Rad real-time thermal cycler CFX96, using the *Wb*-LDR primers [17] and the SYBR Green fluorescence dye with melting curve analysis. The target sequence size is 90 bp long. Each reaction contained 12.5 μl of IQ SYBRGreen supermix 2× (Biorad), 0.75 μl of 10 pmol μl^-1^ of forward and reverse primers and 5 μl of DNA template in a total volume of 25 μl. Thermal cycling conditions for PCR were 95°C for 3 minutes followed by 40 cycles of 10 s at 95°C and 30s at 60°C. The melt curve analysis was performed by reducing the temperature to 55°C for 1 minute and then raising the temperature by increments of 0.5°C every 10 s up to 95°C. The results were analysed with the CFX manager software to calculate the Ct value corresponding to the number of reaction cycles necessary to detect a signal above baseline.

### Assays and results

Before performing the assays with pool samples, the impact of the heat treatment prior to the DNA purification was assessed for each individual sample. To that end, lysates were heated at 100°C for 5 minutes then cooled immediately on ice. DNA purification and qPCR were then performed as described above. The lysates from either 0 mf, 1 mf or 100 mf blood samples were tested in duplicate.

**Figure 1:**
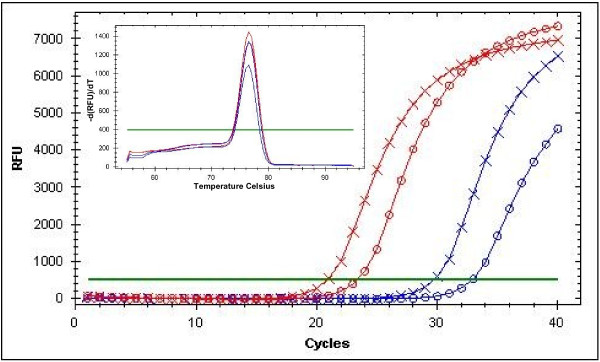
**Amplification and melting curves of LDR real-time PCR (qPCR): impact of the heating step:** Blood samples with 1 mf (blue curves) and with 100 mf (red curves) were tested, The number of reaction cycles needed to detect a signal was lower when the lysate had been heated at 100°C before DNA purification (cross curves) than without this heating step (circle curves). The melt peak temperature was the same for each sample = 76.5°C.

An amplicon of the expected 90 bp size as estimated by electrophoresis was produced from all samples containing mf, with a melt peak temperature of 76.5°C. No amplification was observed for negative samples. For both mf concentrations, the PCR amplification signal was detected earlier in samples that had undergone the heating treatment (Figure 1). We concluded that the heating step improved the outcome of DNA amplification and decided to include this step before DNA purification in all subsequent assays.

Furthermore, we verified that qPCR sensitivity was maintained when processing only a fraction of the lysate. For that purpose, blood samples spiked with one mf and samples having ≈ 100 mf were used. For both mf concentrations, two volumes of lysate were processed: either the whole lysate (600 μl) as described previously [2] or one sixth of the lysate (100 μl). Seven blood samples spiked with one mf were processed, 3 with the whole lysate and 4 using only 100 μl of lysate. Negative blood samples and 100 mf blood samples were analysed in duplicate. Details of samples analysed and average Ct values are reported in Table 1. No PCR amplification was detected for the negative samples. The signal obtained from 100 μl of lysate compared to that from the whole lysate was weaker due to the reduced quantity of DNA processed. Nevertheless, positive amplification using one sixth of 1 mf blood lysate was systematically obtained (average Ct value of 31.9 cycles), showing no loss of sensitivity.

**Table 1:** qPCR results depending on lysate volume processed and on microfilaria (mf) burden

**mf per sample**	**Volume of lysate (μl)**	**Sample replicates**	**Ct mean**	**Ct range**	**Tm**
1	600	3	29.8	[28.6-31.9]	76.5
	100	4	31.9	[31.0-33.1]	76.5
100	600	2	21.6	[21.5-21.6]	76.5
	100	2	23.6	[23.5-23.7]	76.5

Finally, taking those results into account, we combined 100 μl of individual lysates in pools of increasing sizes, with the objective of detecting a single mf positive sample diluted in at least 11 negative samples. The pools were prepared by mixing the lysate of a single mf positive sample with the lysates of 0 to 11 negative blood samples. DNA of mixes was purified and the qPCR performed as described above. Four pools were made and tested for each pool size and the mean Ct values are reported in Table 2. These values obtained ranged between 31.4 and 32.3 thus showing limited variability. We concluded that diluting the *Wb* target DNA in increasing amounts of human DNA did not prevent its amplification. This demonstrates that using the improved qPCR assay allows detection of a single mf from a 60 μl dried blood spot (equivalent to a burden of 18 mf/ml) diluted in a pool of at least 12 samples. Further analyses of large sample size were not tested.

**Table 2:** Ct values depending on lysate volume and lysate number per pool

**Sample volume (μl)**	**Pool size**	**Pools processed**	**Ct mean**	**Range**	**Tm**
100	1	4	31.9	[31–33.1]	76.5
	5	4	31.4	[30.3-32.2]	76.5
	10	4	31.9	[31.1-32.9]	76.5
	12	4	32.3	[31.2-33.7]	76.5
50	10	3	33.3	[32.7-33.7]	76.5
	12	1	33.6		76.5

The experiment was repeated using only 50 μl of lysate per sample to save time by reducing the number of centrifugations necessary during the DNA purification step. We have processed three pools of 10 lysates and one pool of 12 lysates, and obtained mean Ct values of 33.3 and 33.6 respectively (Table 2). The results showed that as little as 50 μl of lysate could be used to constitute the pools without loss of sensitivity.

Using the Ct values obtained with all low positive and negative samples tested, an arbitrary threshold was set at a Ct value of 36 (threshold baseline at 500) to exclude false positive results. All results with a Ct value below 36 and a melt peak temperature of 76.5°C were therefore considered positive.

**Figure 2:**
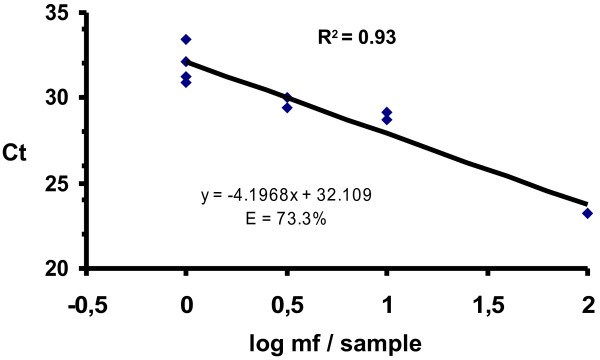
**Quantitative standard curve.** Shown is the curve obtained by LDR qPCR with CFX96 instrument, using DNA from 100 μl of lysate of blood samples spiked with 1, 3, 10 and 100 mf.

*Wb* PCR assays are not currently reported as quantitative. We generated a standard curve using 100 μl of lysate from blood samples with 1, 3, 10 or 100 mf to be able to quantify the qPCR results (Figure 2). There was a linear relationship between the log number of mf spiked and the number of reaction cycles needed to detect signal above baseline. Using this method, 100 μl of each individual lysate contained in an LF positive pool can be tested retrospectively and the mf concentration(s) of infected individuals estimated using the standard curve. This additional information could be helpful for program managers to estimate the risk of transmission, as the number of mf ingested by mosquito vector increases as the density of mf in blood increases.

## Conclusion

One of the principal measures of success of the GPELF is the decrease in microfilaremia prevalence and in mf load, a sine qua non condition to LF elimination. Our improvement of the PCR-based pool screening method allows for a highly sensitive screening tool relevant for LF control. Although this assay currently requires real-time PCR equipment, it may ultimately be adapted for use in resource-poor endemic areas [28]. The present assay may stimulate the development and support of much-needed national and regional reference laboratories to suit the various LF epidemiological situations and populations at risk.

## Competing interests

The authors declare having no competing interests.

## Authors’ contributions

CP conceived and designed the experiments. CP performed the experiments and analyzed the data. CP and AL wrote the paper. Both authors read and approved the final manuscript.
